# Clinical Value of the Assessment of Changes in MEP Duration with Voluntary Contraction

**DOI:** 10.3389/fnins.2015.00505

**Published:** 2016-01-11

**Authors:** Marisa Brum, Christopher Cabib, Josep Valls-Solé

**Affiliations:** ^1^São Bernardo Hospital, Centro Hospitalar de SetúbalSetúbal, Portugal; ^2^Instituto de Medicina Molecular, Faculty of Medicine, University of LisbonPortugal; ^3^EMG Unit, Neurology Department, Hospital Clinic, University of BarcelonaBarcelona, Spain; ^4^Institut d'Investigacions Biomèdiques August Pi i Sunyer, Universitat de BarcelonaBarcelona, Spain

**Keywords:** motor evoked potential, contraction-induced facilitation, stroke, multiple sclerosis, spastic paraparesis, psychogenic weakness

## Abstract

Transcranial magnetic stimulation (TMS) gives rise to muscle responses, known as motor evoked potentials (MEP), through activation of the motor pathways. Voluntary contraction causes facilitation of MEPs, which consists of shortening MEP latency, increasing MEP amplitude and widening MEP duration. While an increase in excitability of alpha motorneurons and the corticospinal tract can easily explain latency shortening and amplitude increase, other mechanisms have to be accounted for to explain the increase in duration. We measured the increase in duration of the MEP during contraction with respect to rest in a group of healthy volunteers and retrospectively assessed this parameter in patients who were examined in a standardized fashion during the past 5 years. We included 25 healthy subjects, 21 patients with multiple sclerosis, 33 patients with acute stroke, 5 patients with hereditary spastic paraparesis, and 5 patients with signs suggesting psychogenic paresis. We found already significant differences among groups in the MEP duration at rest, patients with MS had a significantly longer duration, and patients with stroke had significantly shorter duration, than the other two groups. The increase in MEP duration during voluntary contraction was different in patients and in healthy subjects. It was significantly shorter in MS and significantly longer in stroke patients. It was absent in the five patients with suspected psychogenic weakness. In patients with HSP, an abnormally increase in duration occurred only in leg muscles. Our results suggest that the increase in duration of the MEP during contraction may reveal the contribution of propriospinal interneurons to the activation of alpha motorneurons. This mechanism may be altered in some diseases and, therefore, the assessment proposed in this work may have clinical applicability for the differential diagnosis of weakness.

## Introduction

Transcranial magnetic stimulation (TMS) gives rise to muscle responses, known as motor evoked potentials (MEP), through activation of the motor pathways (Amassian et al., 1987; Day et al., 1987, 1989; Rothwell et al., 1987, 1991). Voluntary contraction causes facilitation of the MEPs, which consists of shortening the MEP latency, increasing the MEP size and widening the MEP duration resulting in an increase in total MEP area (Mills et al., 1987; Valls-Solé et al., 1994a,b). While latency shortening and amplitude increase can be well explained by assuming that voluntary contraction put more motorneurons near the firing threshold, the rationale behind the increase in MEP duration is less clear. On one side, motorneurons should be firing earlier and more synchronously to give rise to the shortening of latency and increase in peak amplitude. However, MEP duration increases beyond the time that the MEP would end if elicited at rest. Therefore, some motorneurons should receive excitatory inputs with latency longer than at rest. This could be due to activation of slow conducting corticospinal fibers or to the generation of excitatory inputs at spinal level (Brasil-Neto et al., 1992; Di Lazzaro et al., 1999, 2001), but it seems contradictory that slow conducting corticospinal fibers are recruited during voluntary contraction. Many sources of inhibition lead to the silent period after synchronized alpha motorneuron firing. After hyperpolarization prevents motorneurons from immediate reactivation and inhibitory inputs from Renshaw cells should reach the motorneuron within few milliseconds (ms) after the peak. Even if some inhibitory activity may be temporarily switched off during contraction, this does not justify the presence of EMG activity at a time when there are no identifiable excitatory inputs.

The EMG activity that follows the MEP during contraction is not usually taken into account in clinical assessment through TMS. The effects of facilitation are usually evaluated by either the onset latency or the size of the MEP. Onset of silent period is usually measured from more stable marks such as the stimulus artifact or MEP latency onset. We argue that measuring the characteristics of such segment of EMG activity might be of some clinical applicability. We reasoned that, when an MEP is elicited during voluntary contraction, inhibitory inputs generated at spinal level or by the descending volleys should counteract the excitatory commands to effectively end the ongoing EMG activity and give way to the silent period. This may take some time and, meanwhile, the EMG activity will not be suppressed. We considered that such “MEPtail,” i.e., the EMG activity that follows the MEP peak and ends at the beginning of the silent period, should indicate the relationship between the strength of the excitatory inputs reaching the alpha motorneurons during voluntary contraction and the inhibitory inputs derived from the synchronized activation of alpha motorneurons at the time of the MEP. Therefore, we determined the extent of contraction-induced facilitation of the MEP in healthy subjects, with special attention to the increase in MEP duration, and performed a retrospective analysis of how such parameter was affected in various neurological disorders involving the motor pathway, i.e., multiple sclerosis (MS), stroke, hereditary spastic paraparesis (HSP), and psychogenic weakness.

## Methods

We performed a retrospective study of the recordings obtained within the last 5 years in healthy subjects and patients in whom we examined facilitation of the MEP in the first dorsal interosseous muscle (FDI), using a standardized method. We included 25 healthy subjects, 21 patients with multiple sclerosis, 33 patients with acute ischemic stroke, 5 patients with hereditary spastic paraparesis, and 5 patients with the clinical diagnosis of probable psychogenic paresis. In 12 healthy subjects and in the 5 patients with hereditary spastic paraparesis, we recorded the MEP also from the tibialis anterior (TA). The overall inclusion criteria were to have consistently elicitable MEPs at rest to cortical stimulation and a complete study carried out by following the protocol summarized below. Patients with multiple sclerosis were all diagnosed according to the McDonald criteria (Polman et al., 2011). They all had signs compatible with mild to moderate involvement of the motor pathway, with a mean Expanded Disability Status Scale of 3.5 (range between 2 and 6) and no one above four for the functional scale on pyramidal signs (Kurtzke, 1983). Patients with stroke were examined within the first 2 weeks after presentation of the lesion and had mild to severe hemiparesis due to a subcortical ischemic middle cerebral artery infarct. Patients with hereditary spastic paraparesis were all genetically mediated, spastin positive (SPG4), each of them belonging to a different family. Patients with psychogenic weakness had all normal diagnostic tests for possible lesions in the motor pathway and clinical evidence of inconsistent weakness, out of proportion of examination findings.

Retrospective data were all collected following a standardized protocol, which contemplated recording at rest and during a voluntary contraction of about 30% of maximum. The stimuli were applied with a Magstim (Magstim Company, Dyfed, UK), equipped with either a figure-of-8 coil for hand muscles or a circular coil for leg muscles. Subjects were sitting, relaxed, and alert. Silver/silver chloride disk electrodes were used for recording all responses. They were attached bilaterally, in a belly-tendon montage, over the FDI for the study of upper limb muscles, which was done in all subjects, and the TA for the study of lower limb muscles in healthy subjects and patients with HSP. EMG signals were filtered and amplified, and traces were recorded using a KeyPointNet electromyograph. Stimulus intensity was fixed at about 120% resting motor threshold, which, based on the recommendations of Rossini et al. (1994), we determined as the minimum stimulus intensity that gave rise to a MEP of at least 50 μV amplitude in at least 50% of trials when TMS was applied to the appropriate scalp location for the target muscle, with the subject at rest. In all instances, we recorded a variable number of MEPs at each stimulation condition (between 2 and 10) and superimposed them at their best fit to facilitate parametric measuring.

In a group of newly recruited 10 healthy subjects, we examined again the effects of facilitation on the MEP to cortical stimulation and added the observation of the effects on the MEP elicited by cervical foraminal stimulation. We asked these subjects to perform two different levels of muscle contraction: mild (10% of maximum voluntary contraction) and strong (30% of their maximum voluntary contraction). At the time of testing, the participants signed an informed consent and the study protocol for retrospective data collection, as well as for the new study in healthy subjects, were approved by the Ethics committee of the Hospital Clinic of Barcelona.

## Data analysis

In each recording, we measured onset latency at the time that the EMG activity became consistently more than 10% above background amplitude, whether at rest or during contraction. Amplitude was measured from the maximum negative peak to the maximum positive peak. Total MEP duration was measured from MEP onset latency to the time at which the activity returned to baseline. We determined the percentage change for each MEP parameter during contraction with respect to rest. For the statistical comparison among groups, we chose to analyze the recordings from the dominant side in healthy subjects and the most impaired side in patients. In the 10 newly recruited subjects, we examined the effects of level of muscle contraction (mild and strong) on the MEP onset latency, peak-to-peak amplitude, and duration. We compared also the effects of cortical to those of foraminal stimulation. The outcome measure in which we focused our study was the increase in duration that takes place during contraction at the tail of the MEP (tail), as a specific aspect of MEP facilitation. This was measured in ms as the difference between the end of the MEP obtained during contraction and the end of the MEP obtained at rest.

All data were analyzed using SPSS 21.0 (IBM UK, London). We used repeated measures ANOVA for the analysis of the effects of level of muscle contraction in healthy subjects (rest, mild contraction and strong contraction). A One-way analysis of variance (ANOVA) was used to determine whether there were significant differences in retrospective data among the three independent groups with a sizeable number of subjects (healthy controls, multiple sclerosis, and stroke patients). In the other 2 groups of patients (HSP and psychogenic patients) the sample was too small to do the analyses. *P* ≤ 0.05 was considered statistically significant.

## Results

The newly recruited subjects were 5 men and 5 women, with an age ranging from 32 to 65 (44 ± 7). In the subjects recruited retrospectively, mean age was significantly higher in patients with stroke than in the other groups (Table 1). Data were available from all subjects initially recruited, with no data gaps or missing information.

**Table 1:** **Demographic and general clinical characteristics of patients included in the retrospective study**.

	**Stroke**	**MS**	**HSP**	**Psychogenic**
Age	67 (6.4)^a^	52 (3.1)	49 (4.3)	54 (2.4)
Gender (M/F)	21/12	11/10	4/1	5/0
Weakness (MCR)	1–4	2–5	4–5^*^	0–3^**^
Most imparied side	14 R/19 L	14 R/7 L	Bilateral^*^	4R/1L

a significantly higher than in the other groups.

* In leg muscles.

** As for the patients first expression, with no encouragement.

### MEPs from the FDI to cortical and foraminal stimulation in healthy subjects

**Figure 1:**
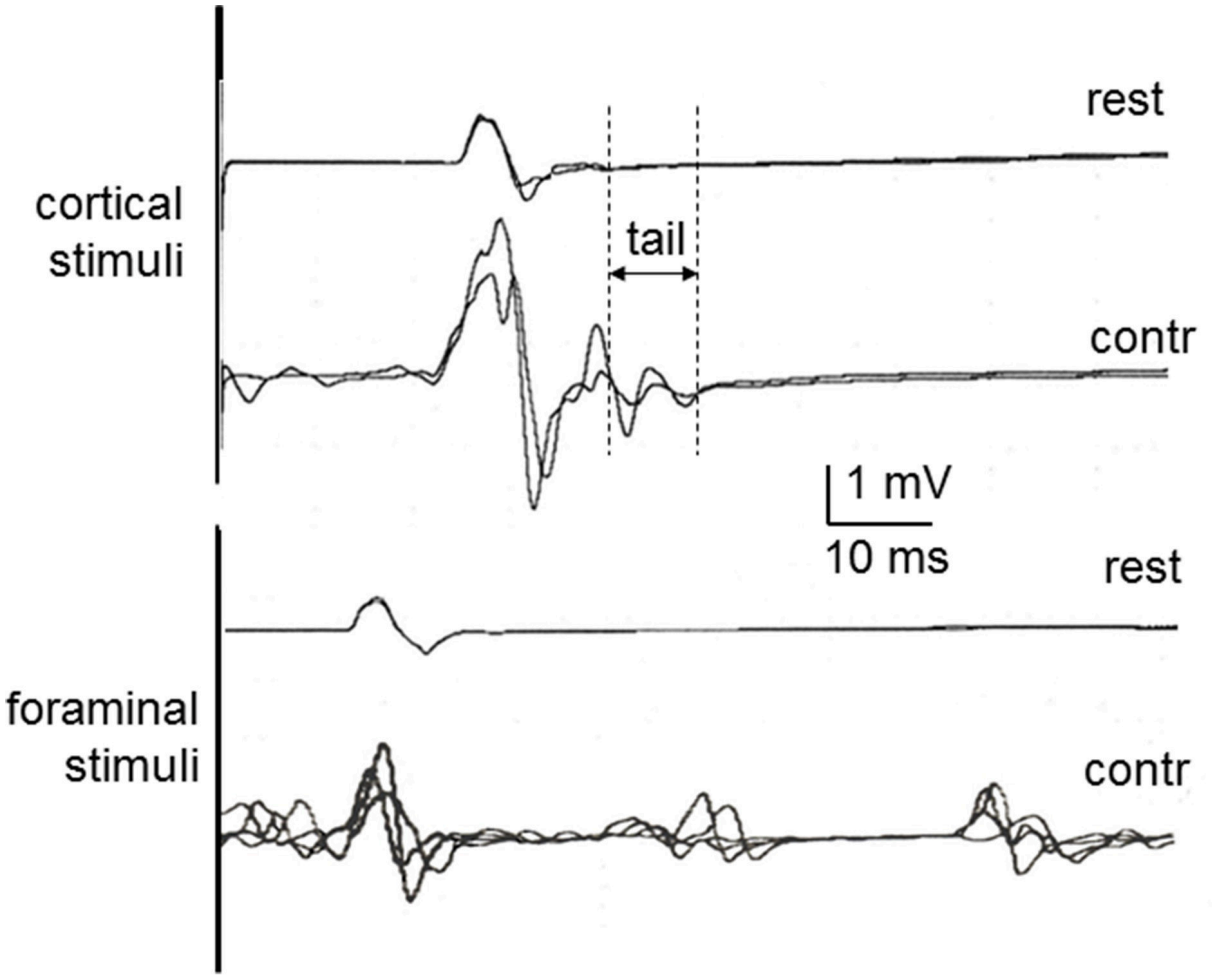
**MEPs in healthy control subjects at rest and during contraction, recorded in the first dorsal interosseous muscle to cortical stimulation (above) and cervical foraminal stimulation (below)**. Rest, Recorded at rest; Contr, Recorded during contraction. The vertical lines illustrate the methods used to measure the MEP tail as one of the aspects of MEP facilitation with contraction.

Representative examples of recordings in one of the newly recruited healthy subject are shown in Figure 1 to cortical and foraminal stimulation. A summary of the mean data is reported in Table 2. The effects of contraction on the MEP elicited with cortical stimulation were the expected ones: shortening of onset latency, increase in amplitude and increase in duration. The repeated measures ANOVA for values obtained when comparing the three conditions (rest, mild contraction, and strong contraction) showed statistically significant differences [ANOVA; *F*_(2, 27)_ = 4.981; *p* = 0.004]. The *post-hoc* analysis showed that all significant differences were found when comparing contraction to rest (*p* < 0.05 for latency, amplitude and duration), but there were no significant differences between data obtained with mild and strong contraction (*p* > 0.05 for all comparisons). The MEP tail increased a mean of 4.1 ms (*SD* = 0.8 ms) with mild contraction and 4.4 ms (*SD* = 0.5 ms) with strong contraction (*p* > 0.05). With foraminal stimulation there were no significant changes in onset latency, amplitude or duration, although amplitude, and duration increased in a few trials in some subjects (Table 2). A burst was consistently seen at a mean latency of 38.7 ms (*SD* = 5.1 ms), interrupting the post-MEP silent period.

**Table 2:** **Data gathered from 10 newly recruited healthy volunteers on facilitation of the MEP with mild and strong voluntary muscle contractions**.

	**Rest**	**Mild**	**%**	**Strong**	**%**
**CORTICAL STIMULATION**					
Onset latency (ms)	21.5 (1.8)	19.7 (1.3)^*^	91.6	19.6 (1.1)^*^	91.1
Amplitude (mV)	1.1 (0.6)	4.1 (0.7)^*^	372.7	4.4 (0.9)^*^	401.0
Duration (ms)	12.7 (2.0)	17.7 (3.3)^a^	139.3	17.9 (3.9)^*^	140.9
**FORAMINAL STIMULATION**					
Onset latency (ms)	13.4 (1.1)	13.3 (1.1)	99.2	13.3 (1.0)	99.2
Amplitude (mV)	0.9 (0.5)	1.3 (0.6)^*^	144.4	1.3 (0.7)^*^	144.6
Duration (ms)	8.4 (1.7)	8.8 (2.1)	104.7	8.8 (2.2)	104.8

*Data are represented as mean and 1 standard deviation (within parenthesis). Data in the columns labeled % are the ratio between contraction and rest for mild and strong contractions. Asterisks refer to a significant difference (p < 0.05) with respect to rest, in the post-hoc analysis after one factor ANOVA*.

### Comparison of data among groups

**Figure 2:**
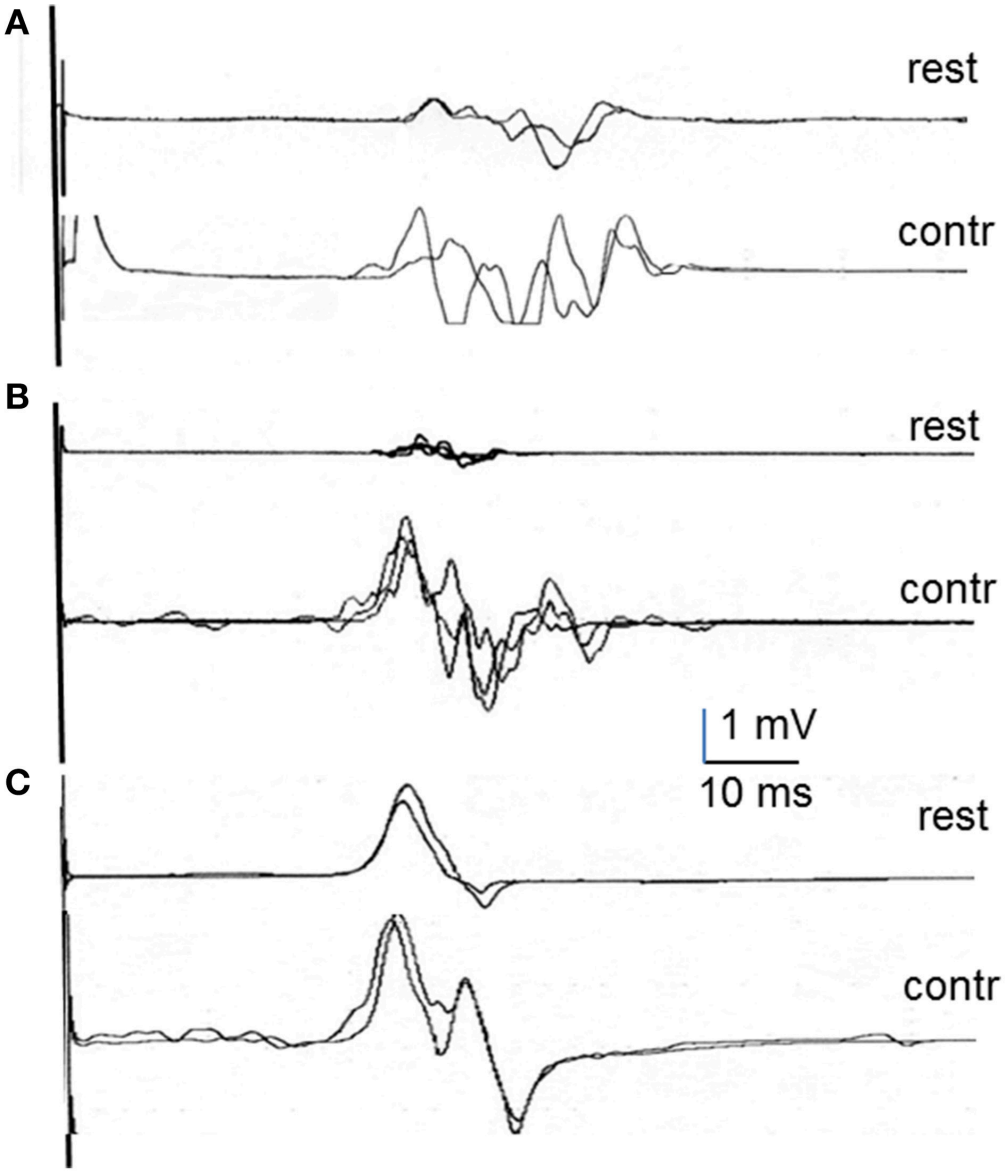
**Examples of MEP recordings in first dorsal interosseous at rest and during contraction in multiple sclerosis patients (A), stroke patients (B), and psychogenic weakness patients (C)**.

Table 3 shows the summary of retrospective data gathered for all groups on the MEP at rest and during contraction as well as the percentage change observed during contraction with respect to rest for onset latency, peak amplitude and MEP duration. Representative recordings of MEPs at rest and during contraction are shown in Figure 2 for the FDI recordings in each group of patients. There were already significant differences among groups in the characteristics of the MEPs recorded at rest. The repeated measures one-factor ANOVA, run only on data from the three groups with a sizeable number of subjects (healthy subjects, stroke, and MS patients), showed significant differences in onset latency, peak amplitude, and MEP duration [*F*_(2, 76)_; *p* < 0.01 for all of them]. The *post-hoc* analyses showed that patients with MS and stroke had delayed onset latency and smaller peak amplitude than healthy subjects (*p* < 0.05 for both groups of patients in both comparisons). In regard to MEP duration, patients with MS had a significantly longer duration, while patients with stroke had significantly shorter duration, than the other two groups (*p* < 0.005 for all comparisons).

**Table 3:** **Mean data on MEPs obtained at rest in the healthy subjects and patients included in the retrospective study**.

	**Onset latency**		**Percentage**	**Peak amplitude**		**Percentage**	**MEP duration**		**Percentage**
	**Rest**	**Contr**		**Rest**	**Contr**		**Rest**	**Contr**	
HV (25)	21.3 (1.9)	19.2 (1.4)	90.14	3.1 (0.7)	6.8 (2.7)	219.35	13.1 (2.3)	17.8 (2.3)	135.88
Stroke (33)	23.6 (3.1)^a^	20.5 (2.2)	86.86	0.7 (1.3)^b^	2.2 (3.1)^b^	307.14	9.3 (4.8)^b^	18.2 (3.1)	195.70^a^
MS (21)	25.5 (4.2)^a^	24.5 (3.3)^a^	96.08^c^	1.4 (0.8)^b^	1.9 (2.2)^b^	135.71^d^	19.4 (2.5)^a^^,^^d^	21.7 (3.7)	111.86^d^
Psychogenic (5)	21.1 (2.2)	20.3 (3.0)	96.21	1.7 (2.3)	2.3 (3.5)	135.29	14.0 (2.1)	14.3 (2.3)	102.14
HSP (5)	22.0 (3.1)	19.9 (2.8)	90.45	2.3 (1.5)	4.8 (3.0)	208.70	14.5 (2.9)	19.1 (4.1)	131.72
HV TA (12)	30.9 (2.8)	28.5 (2.0)	92.23	1.4 (1.0)	2.9 (2.3)	207.14	19.7 (4.5)	29.4 (5.6)	144.16
HSP TA (5)	35.8 (4.7)	35.4 (4.1)	98.88	0.5 (1.2)	0.6 (1.8)	115.38	35.9 (8.4)	36.2 (9.9)	100.83

*Data are the mean and one standard deviation (within parenthesis) measured on the MEP recorded in the first dorsal interosseous (except for HV TA and HSP TA, which were recorded in the tibialis anterior). HV, Healthy volunteers; MS, Multiple sclerosis; HSP, Hereditary spastic paraparesis. The number in parenthesis in the first column refers to the number of patients included in the analysis*.

*Onset latency and duration are measured in ms; peak amplitude in mV. Percentage refers to the percentage of the MEP characteristics during contraction with respect to rest. Superindices a, b, and c refer to significant differences (p < 0.05) found in the comparison among the three groups with a sizeable sample (healthy subjects, patients with stroke, and patients with multiple sclerosis)*.

a *Significantly longer than in healthy subjects*.

b *Significantly shorter than in healthy subjects*.

c *Significantly longer/larger in MS than in stroke*.

d *Significantly smaller/shorter in MS than in stroke*.

Muscle contraction induced facilitation to different degrees in healthy subjects and patients (Table 3). As expected, the percentage shortening in onset latency, increase in peak amplitude, and lengthening in MEP duration, including the increase in MEP tail, calculated in healthy subjects were all similar to those reported above on the newly recruited subjects. The effects of contraction on the MEP in patients were not uniform, and the percentage change with respect to rest showed significant differences in all comparisons [ANOVA; *F*_(2, 76)_; *p* < 0.05]. The *post-hoc* analyses indicated a significantly bigger percentage of onset latency shortening, and a significantly larger percentage of peak amplitude increase, in stroke than in MS patients. In MEP duration, differences were found between groups in all comparisons: There was a smaller increase in MS patients than in healthy subjects and stroke patients, and a bigger increase in stroke patients than in healthy subjects and MS patients (*p* < 0.05 for all comparisons). No significant differences with respect to healthy subjects were observed in hand muscles in patients with HSP but they were observed in leg muscles (reported below). In patients with psychogenic weakness, MEP duration was similar at rest and during voluntary contraction, although a moderate shortening of latency and increase in amplitude was observed in some traces.

A more striking difference among groups was observed in the MEP tail (Table 4). The contraction-induced increase in MEP duration beyond the end of the MEP at rest was significantly different among most groups, including psychogenic patients, in the FDI.

**Table 4:** **Differences in MEP tail among groups of subjects**.

**Recording**	**Group (N)**	**Increase in MEP tail**
FDI	Control (25)	4.7 (0.7)
	MS (21)	1.4 (1.2)^a^
	Stroke (33)	8.5 (1.8)^b^
	Psychogenic (5)	0.8 (2.1)^a^
	HSP (5)	4.5 (0.9)
TA	Control (12)	6.1 (1.1)
	HSP (5)	0.5 (0.9)^a^

*Data are the mean calculated among all subjects of the same group. Superindices a and b refer to the statistical significance measured with the post-hoc comparison after one-factor ANOVA including all groups recorded in the same muscle*.

a *Significantly shorter than in control subjects*.

b *Significantly longer than in all other groups*.

### MEPs from the tibialis anterior

**Figure 3:**
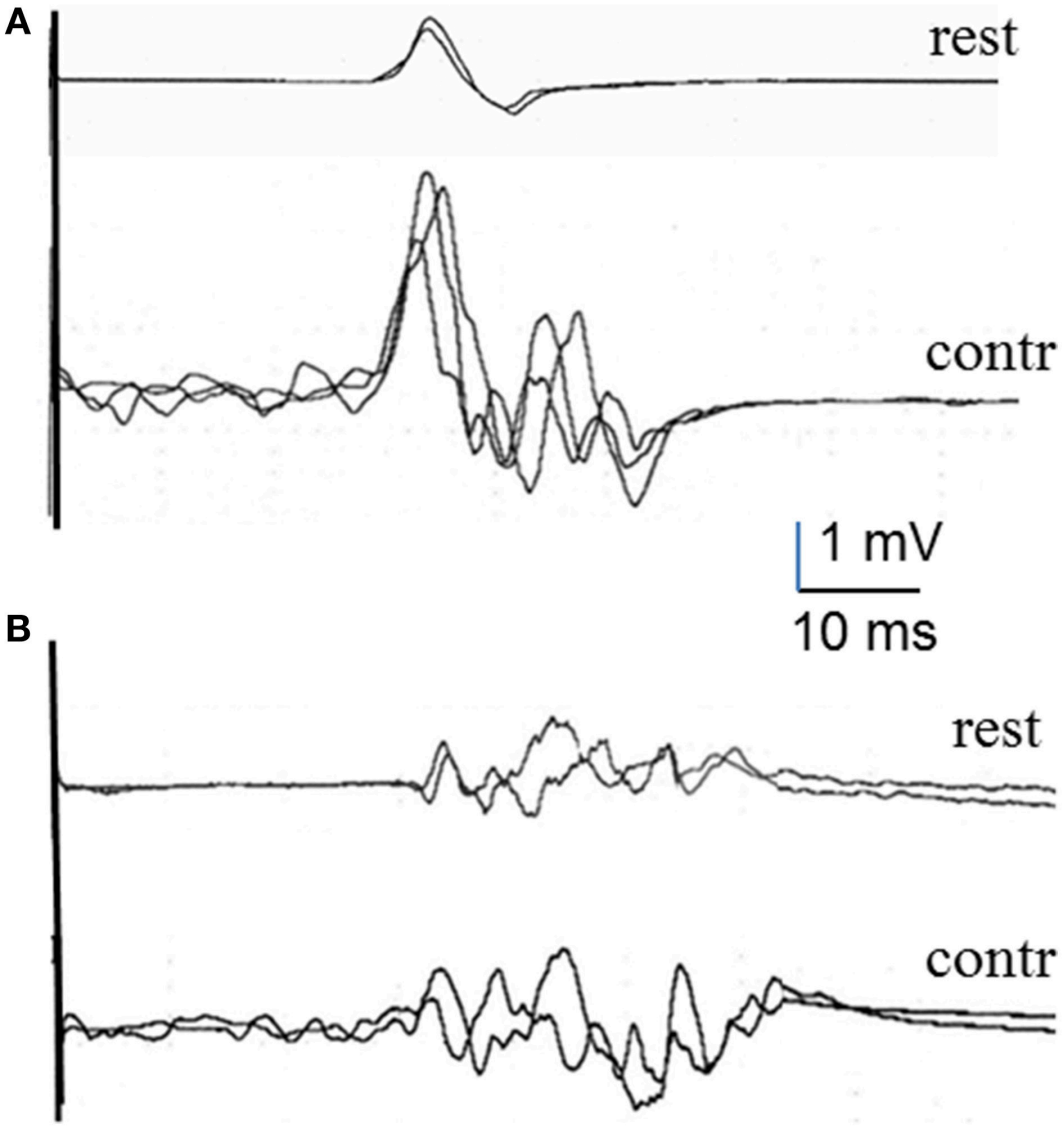
**MEPs recorded in the tibialis anterior muscle at rest and during voluntary contraction in a healthy subject (A) and an HSP patient (B)**.

Data were gathered from 12 healthy subjects and the 5 patients with HSP. Although we did not run comparative statistics because of the small number of subjects, differences between the two groups of subjects were already clear at rest (Table 3) and more so in the percentage increase of MEP duration with voluntary contraction. The mean MEP tail increase was markedly larger in healthy subjects than in patients (Table 4), with a significant difference in the unpaired *t*-test (*p* < 0.05). Figure 3 shows representative TA recordings from healthy controls and patients with HSP.

## Discussion

The main physiological mechanism for MEP facilitation is, likely, the increase in spinal motorneuronal excitability. During voluntary contraction, the descending volley will meet with more motoneurons available for firing than at rest (Di Lazzaro et al., 2008). Descending volleys after cortical stimulation in humans have been recorded with epidural electrodes (Di Lazzaro et al., 1999). While a direct (D) wave can be obtained with electrical stimulation, this is usually not so with TMS (Day et al., 1987; Di Lazzaro et al., 1999), where mostly indirect (I) waves are recorded. The I waves have an interpeak latency of about 1.2–2.0 ms, up to about 6 ms after the D wave (Day et al., 1987; Boniface et al., 1991; Nakamura et al., 1997). The ultimate target of such descending volleys is assumedly the alpha motorneuron, but an unknown number of excitatory and inhibitory propriospinal interneurons may lay in between. The number of descending I waves increases little when subjects perform a voluntary contraction (Di Lazzaro et al., 1999). Therefore, the large increase in the MEP duration reveals that facilitation takes place mostly because of summation of inputs from various sources at the alpha motorneuronal level.

Polyphasic MEPs have been reported so far in various diseases. In patients with ALS (Kohara et al., 1999), the authors suggested that polyphasic MEPs were due to activation of additional motor pathways, such as slow monosynaptic pyramidal or even polysynaptic pathways in ALS patients. In patients with DYT11-positive myoclonus-dystonia syndrome (van der Salm et al., 2009), the authors hypothesized that the mutation associated with the disease, SGCE gene, could have caused changes in membrane properties or ion channels, leading to asynchronous discharge timing of spinal motorneurons by the descending corticospinal activity. MEP polyphasia has been also reported in MS, who have already an increase in MEP duration at rest (Kukowski, 1993), compatible with increased temporal dispersion of the impulses reaching the spinal motorneurons. Recently, in patients with idiopathic generalized epilepsy (IGE) and first-degree relatives, Chowdhury et al. (2015) showed increased polyphasia, attributed to abnormal timing and patterning of the descending volleys in the corticospinal tract, and suggested that this was a novel endophenotype in this pathology. No data are available on changes in MEP duration in patients so far and, specifically in the increase in the MEP tail, beyond the end of the MEP at rest. We found that this parameter was the one discriminating better among our groups of subjects.

Patients with MS and HSP had longer duration of MEP already at rest, with little increase during contraction. In patients with stroke, if their damaged corticospinal tract was still excitable, the MEP increased significantly in amplitude and duration, indicating preservation of spinal mechanisms involved in contraction-induced facilitation. The only group studied in which we found no increase in duration was in the patients with psychogenic weakness. This was probably due to voluntary absence of energization of alpha motorneurons by these patients.

The mechanisms involved in the increase in MEP duration with muscle contraction are not clear but our results are consistent with a role for the excitatory pre-motor interneurons that are activated by descending inputs from contralateral corticospinal tract. Patients with MS and HSP, with lesions in the spinal cord, had increased duration at rest likely because of dispersion of the descending volley reaching the alpha motorneuron. These patients could not increase the synchronization of their motorneuronal firing, and the possible implication of the interneurons is masked by the already long duration of the MEP. In patients with stroke, whose spinal interneurons are not altered, the mechanisms of contraction-induced facilitation are fully activated, provided that the volley reaches the spinal level. In opposition, patients with psychogenic weakness do not perform the maximum voluntary contraction when requested, and may not set their spinal interneurons excitability at the level required for facilitation of the MEP during contraction.

The main limitation of our study is that it is retrospective and, therefore, it was not specifically designed for the study of the effects of duration. Even though the data reported here were generated after standardized recordings with no missing values, replication of the study is needed before firm conclusions. Meanwhile, we can conclude that the increase in duration of the MEP during contraction beyond the end of the resting MEP may reveal the activation of premotor propriospinal interneurons by descending inputs. This mechanism may be altered in some diseases. The patterns of MEP facilitation with voluntary contraction may differ depending on the disease, and the study of this feature can be of some clinical utility in the differential diagnosis of weakness.

## Author contributions

MB: Review of patients' reports, Analysis of data, and Writing the first draft. CC: Analysis of data, Statistical analysis, and Revision of the manuscript. JV: Conceptual design of the study and Revision and finalization of the manuscript.

## Conflict of interest statement

The authors declare that the research was conducted in the absence of any commercial or financial relationships that could be construed as a potential conflict of interest.
